# Data on the vegetative response of cowpea to fertilizer application on three selected benchmark soils of the Upper West region of Ghana

**DOI:** 10.1016/j.dib.2020.105590

**Published:** 2020-04-20

**Authors:** Obianuju Chiamaka Emmanuel, Olayiwola Akin Akintola, Francis Marthy Tetteh, Olubukola Oluranti Babalola

**Affiliations:** a Food Security and Safety Niche area, Faculty of Natural and Agricultural Sciences, North-West University, 2735 Mmabatho, South Africa; b Farming Systems Programme, National Horticultural Research Institute, Ibadan, Nigeria; c Soil Research Institute, Kwadaso, Kumasi, Ghana

**Keywords:** Biomass, Crop residue, Soil fertility, Soil organic matter, Yield

## Abstract

Declining soil fertility among smallholder farmers in the Savannah zones of Ghana, among other issues, is triggered by continuous cultivation, low fertilizer use and low soil organic matter content. The area is faced with insufficient domestic production, food insecurity and poverty, all of which constitute major constraints to national development. Continuous cultivation leads to low soil organic matter levels. To build up the soil organic matter levels, residue incorporation is a major factor to be considered. Cowpea is grown in these areas for the grain yield while the residue is incorporated into the soil to gain maximum benefits of the nitrogen fixation. We present the physical and chemical properties of three benchmark soils in the Savannah zones of Ghana as well as their vegetative response to NPK fertilizer application. The FAO soil classification also helps in the thorough understanding of the soil and an appropriate management option for optimal productivity is recommended.

Specifications table

**Table:** 

**Subject**	Agriculture
**Specific subject area**	Soil classification, plant nutrition, fertilizer use
**Type of data**	Table, Graph and Figure
**How data were acquired**	Ten fertilizer treatments were applied on cowpea to determine the effect on vegetative yield of cowpea. The plot size was 24 m^2^ with 4 replications giving a total of 40 plots per soil type. Data were collected and analyzed using Genstat 11th edition. Soil classification was based on FAO WRB (2014) [1].
**Data format**	Raw, analyzed.
**Parameters for data collection**	Soil profile characterization, as well as standard laboratory protocols, was used for soil chemical and physical analysis. Vegetative growth data (height, number of leaves and leaf area) was collected at two, four and six weeks after sowing using a tape measure and leaf area meter
**Description of data collection**	10 random representative crop stands for each treatment were selected for the collection of plant height, the number of leaves and leaf area. The profile pits were classified by determining the parent material, drainage class, horizon distinction and depth of horizons, soil consistency and color through field observation. Munsell color chart was used to determine the soil color.
**Data source location**	City/Town/Region: Dondori- Lawra, Upper West region
	Country: Ghana
	Coordinates (Latitude and longitude) for sampling location: N 10^o^ 40^’^ 13.3^”^ W 002^o^ 51’ 14.8”
	City/Town/Region: Kojopere- Nadowli, Upper West region
	Country: Ghana
	Coordinates (Latitude and longitude) for sampling location: N 10^o^ 20^’^ 40.6^”^ W 002^o^ 13^’^ 44.7^”^
	City/Town/Region: Nyoli - Wa West, Upper West region
	Country: Ghana
	Coordinates (Latitude and longitude) for sampling location: N 09^o^ 46’ 16.0” W 002^o^ 30’ 52.1”
**Data accessibility**	Raw data were deposited at repository Mendeley data; Dec 18, 2019
	Direct URL to data: http://dx.doi.org/10.17632/xmxkt726vs.1

## Value of the data

• This dataset will be valuable for researchers, extension workers and farmers in choosing appropriate soil management practices for optimal and sustainable crop production in the Upper West region of Ghana.
• The data will allow researchers and farmers to determine the suitability of the soil to support the production of cowpea and other legumes in general. The information may also be used by the Government to formulate an appropriate policy and intervention strategies for increased food production for the growing population.
• The data provide insight on the effect of fertilizer treatments and soil types on the vegetative growth of cowpea. Cowpea requires balanced nutrition of nitrogen, phosphorus and potassium for vegetative growth. The vegetative part of the crop forms the biomass that is returned to the soil which helps in the buildup of soil organic carbon.
• This data will be a guide to farmers in the investigated areas on the best fertilizer combination for optimum performance of cowpea. It will also be a guide for long term investigation into soil nutrient dynamics for sustainable cowpea production in the investigated regions.

## 2 Data description

**Fig. 1:**
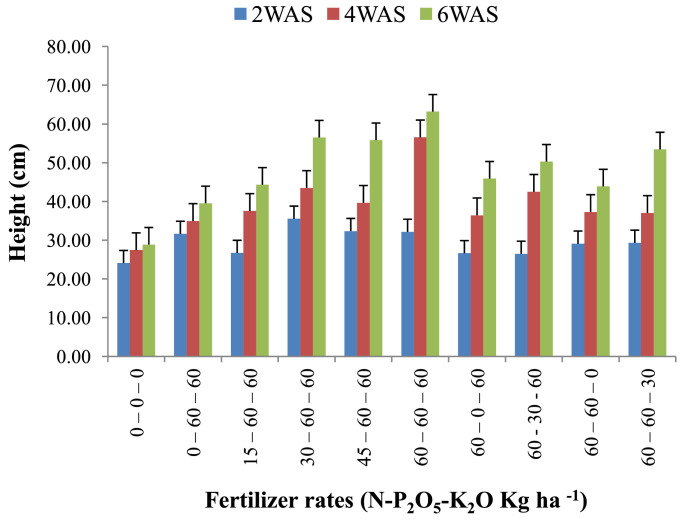
Fertilizer treatment effects on the height of cowpea at Dondori. Bars represent LSD values, WAS – Weeks after sowing.

**Fig. 2:**
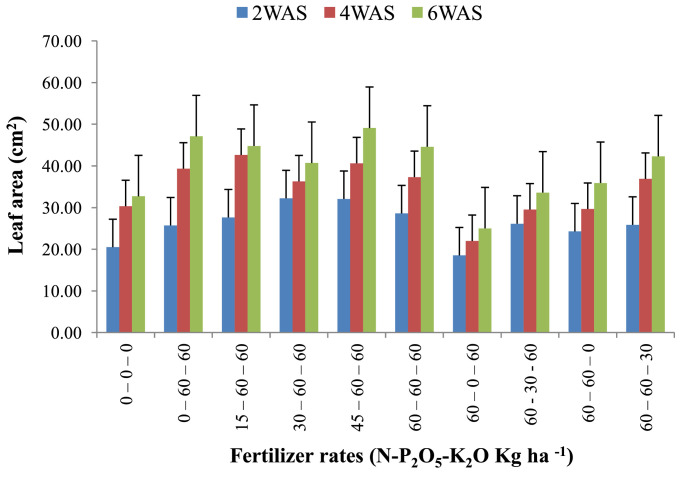
Fertilizer treatment effects on leaf area of cowpea at Dondori. Bars represent LSD values, WAS – Weeks after sowing.

**Fig. 3:**
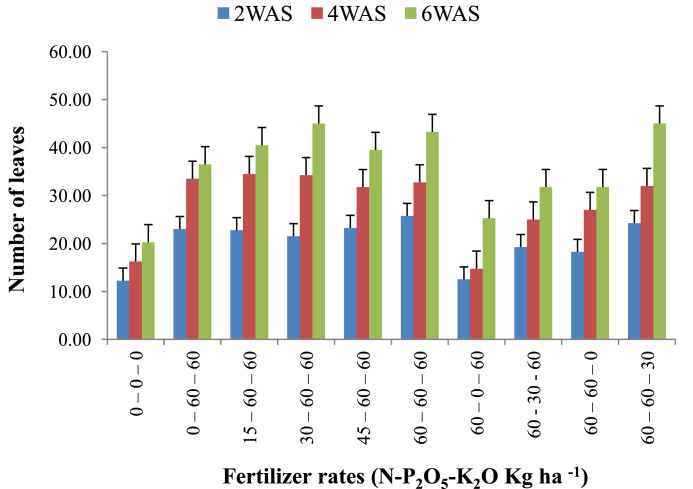
Fertilizer treatment effects on the number of leaves of cowpea at Dondori. Bars represent LSD values, WAS – Weeks after sowing.

**Fig. 4:**
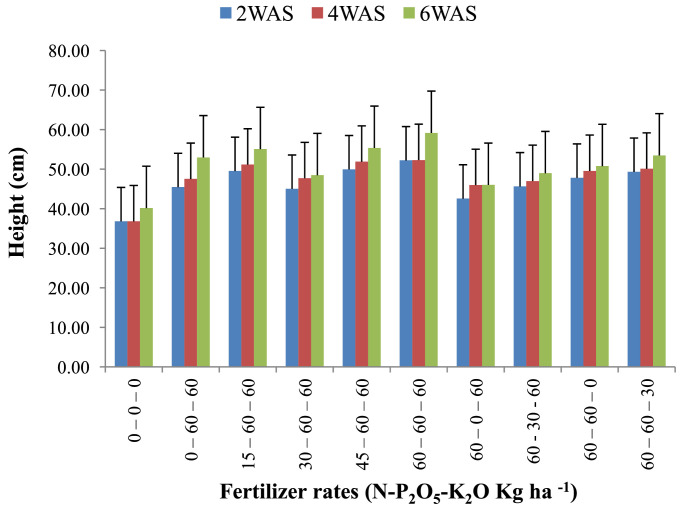
Fertilizer treatment effects on the height of cowpea at Nyoli. Bars represent LSD values, WAS – Weeks after sowing.

**Fig. 5:**
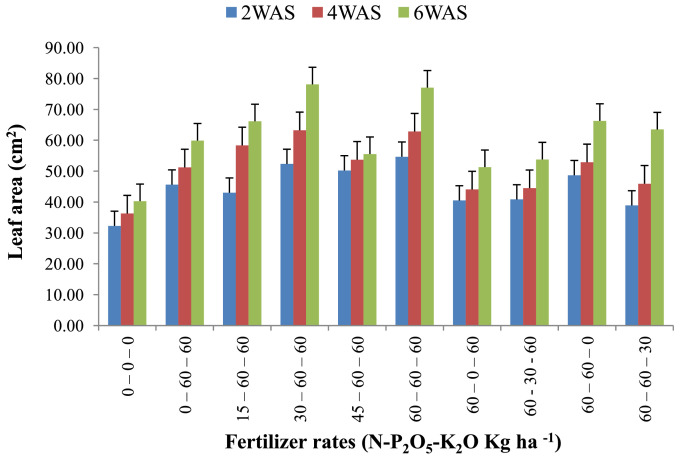
Fertilizer treatment effects on leaf area of cowpea at Nyoli. Bars represent LSD values, WAS – Weeks after sowing.

**Fig. 6:**
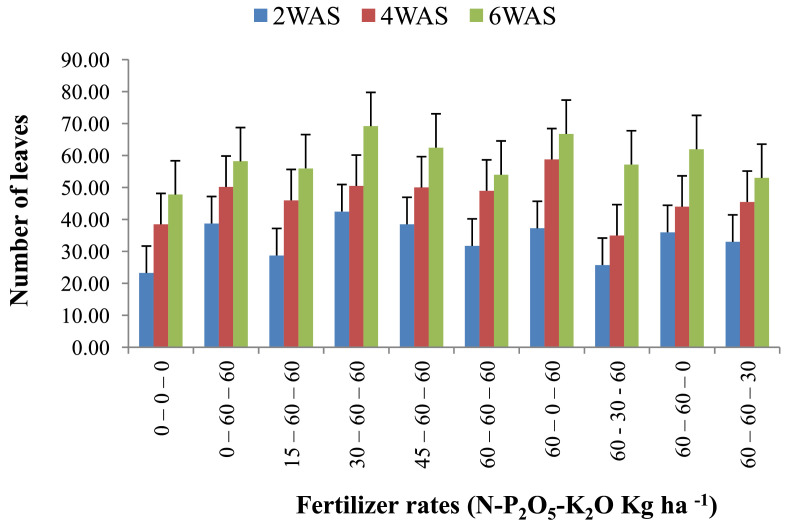
Fertilizer treatment effects on the number of leaves of cowpea at Nyoli. Bars represent LSD values, WAS – Weeks after sowing.

**Fig. 7:**
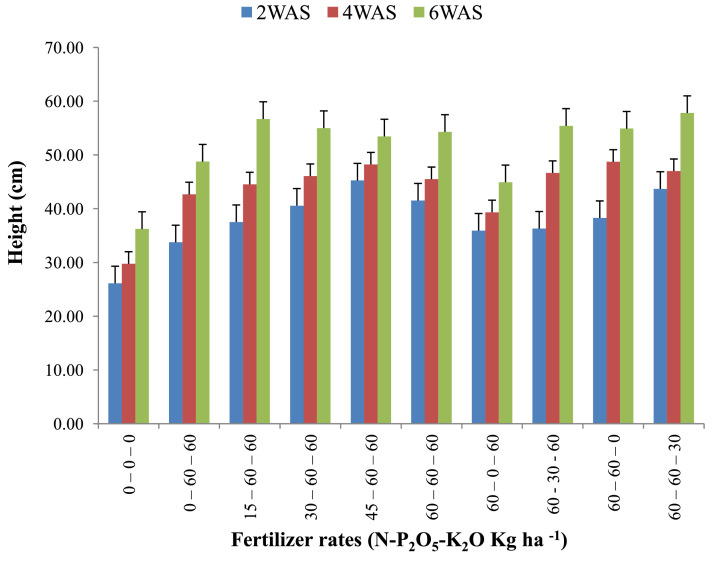
Fertilizer treatment effects on the height of cowpea at Kojokpere. Bars represent LSD values, WAS – Weeks after sowing.

**Fig. 8:**
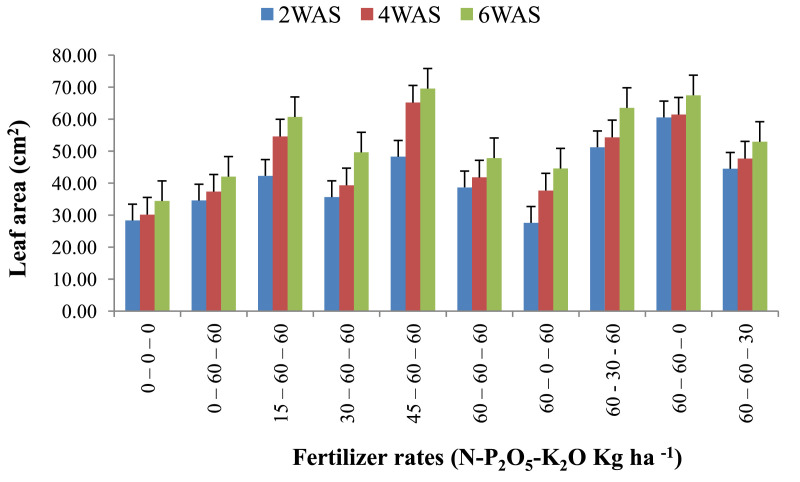
Fertilizer treatment effects on leaf area of cowpea at Kojokpere. Bars represent LSD values, WAS – Weeks after sowing.

**Fig. 9:**
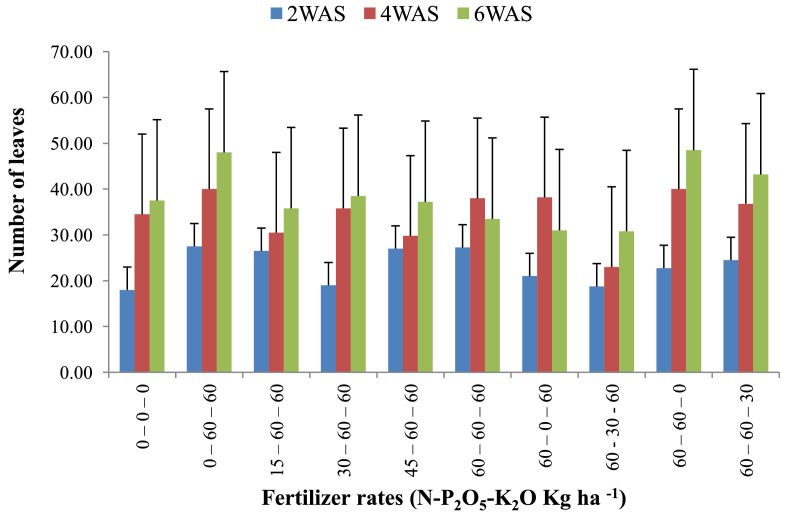
Fertilizer treatment effects on the number of leaves of cowpea at Kojokpere. Bars represent LSD values, WAS – Weeks after sowing.

The data are from 40 experimental plots in each of the 3 sites located in each of the investigated soil types. This was collected from three benchmark soils of the Upper West region of Ghana, West Africa. This region is of utmost importance as it is part of the “breadbasket” region of Ghana. The soil profile differentiations and characteristics observed in the field for the three soils (Dondori, Kojokpere and Nyoli) are presented in Tables 1–3, respectively. It shows the horizon descriptions, depth, drainage and FAO classification of each soil. The physical and chemical properties of the soil profile pit at different depths for each location are presented in Table 4. The Horizon depths for Kojokpere was 0–19, 19–44, 44–88, 88–121 and 121–172 cm. That of Dondori was 0–15, 15–32, 32–91, 91–133, 133–180 cm while Nyoli was 0–12, 12–32, 32–65, 65–82, 82–120. The effect of fertilizer treatments on cowpea height, leaf area and the number of leaves at Dondori are presented in Figs. 1–3, respectively. Figs. 4–6 show the fertilizer treatments effects on cowpea height, leaf area and the number of leaves at Nyoli. The treatment effects on cowpea height, leaf area and the number of leaves at Kojokpere are presented in Figs. 7–9, respectively. The data are available in the repository [2].

**Table 1.** Soil profile Pit characterization of Dondori - Lawra.

Horizon	Depth (cm)	Descriptions
Ap	0 - 15	Strong brown (7.5Yr 4/6); sandy loam; few quartz stones; weak granular; occasional ironstone concretions; many very fine and fine roots; clear and smooth boundary.
BA	15 - 32	Brown (7.5Yr 5/4); sandy loam; few quartz gravels and stones; weak crumbs; frequent ironstones and MnO_2_ concretions; few fine roots; clear and smooth boundary.
Btcs_1_	32 - 91	Yellowish red (5Yr 4/6); dark red (2.5Yr 3/6) mottle; sandy clay loam; frequent quartz gravels sandstones; moderately medium sub-angular blocky; frequent ironstone concretions; very few fine roots; animal burrow; clear and smooth boundary.
Btcs_2_	91 - 133	Yellowish red (5Yr 5/6) dark red (2.5Yr 4/8) mottle; sandy clay loam; common quartz gravels and stones; moderately medium sub-angular blocky; frequent ironstones and MnO_2_ concretions; clear and smooth boundary.
Btcs_3_	133 - 180	Yellowish red (5Yr 5/6); dark red (2.5Yr 4/8) mottle; clay loam; common quartz gravels and stones; moderately medium sub-angular blocky; common ironstone and MnO_2_ concretions.

Coordinates N 10^o^ 40^’^ 13.3^”^ W 002^o^ 51’ 14.8”

Series: Dorimon

Location: Dondori- Lawra

Vegetation: Sudan Savanna

Site: Middle Slope

Parent Material: Lower Birrimian Rock

Drainage: Moderately Well Drained

Classification: Ferric Lixisol

**Table 2.** Soil profile Pit characterization of Kojokpere - Nadowli.

Horizon	Depth (cm)	Description
Ap	0 - 19	Dark brown (10Yr 3/3); coarse sandy loam; few quartz gravels and stones; weak crumbs; occasional ironstone and MnO_2_ concretions; many very fine and common coarse roots; clear and smooth boundary.
AB	19 - 44	Dark yellowish brown (10Yr 3/4); coarse sandy loam; common quartz gravels and stones; weak crumbs; frequent ironstones and MnO_2_ concretions; many fine and common coarse roots; clear and smooth boundary.
Btcs_1_	44 - 88	Brown (7.5Yr 4/4); coarse sandy loam; common quartz gravels and few quartz stones; moderately medium sub-angular blocky; frequent ironstone and MnO_2_ concretions; many fine and common coarse roots; clear and smooth boundary.
Btcs_2_	88 -121	Strong brown (7.5Yr 4/6); coarse sandy loam; common quartz gravels and stones; loose; abundant ironstones and MnO_2_ concretions; few fine and few coarse roots; clear and smooth boundary.
Btcs_3_	121 - 172	Brown (7.5Yr 5/4); clay loam; abundant quartz gravels and many quartz stones; loose; abundant iron and MnO_2_ concretions; few coarse roots.

Series: Kolingu

Coordinates N 10^o^ 20^’^ 40.6^”^ W 002^o^ 13^’^ 44.7^”^

Location: Kojopere- Nadowli

Vegetation: Guinea Savanna

Site: Middle Slope

Parent Material: Cape Coast Granite

Drainage: Well Drained

Classification: Chromic Luvisol

**Table 3.** Soil profile Pit characterization of Nyoli – Wa West.

Horizon	Depth (cm)	Descriptions
Ap	0 - 12	Dark brown (10Yr 3/3); fine sandy loam; few quartz gravels and very few quartz stones; weak fine granular; few ironstones and MnO_2_ concretions; abundant very fine many fine common medium and few coarse roots; clear and smooth boundary.
AB	12 - 32	Dark yellowish brown (10Yr 3/6); fine sandy clay loam; few quartz gravels and stones; moderately medium sub-angular blocky; frequent ironstone and MnO_2_ concretions; many very fine many fine common medium and common coarse roots; clear and smooth boundary.
Btcs	32 - 65	Strong brown (7.5Yr 4/6); light olive brown (2.5Yr 5/6) mottle; clay loam; few quartz gravels and very few quartz stones; moderately medium sub-angular blocky; abundant ironstone and MnO_2_ concretions; very few very fine few fine and few medium roots; clear and smooth boundary.
Btcs_1_	65 -82	Strong brown (7.5Yr 4/6); olive-yellow (2.5Yr 6/6) mottle; clay loam; very few quartz gravels and stones; moderately medium sub-angular blocky; abundant ironstones and MnO_2_ concretions; few fine and few coarse roots; clear and smooth boundary.
Btcs_2_	82 - 120	Brown (7.5Yr 5/4) dark red (2.5Yr 4/8) mottle; clay loam; very few quartz gravels and stones; moderately medium sub-angular blocky; abundant ironstones and MnO_2_ concretions; very few medium roots.

Series: Varempere

Coordinates N 09^o^ 46^’^ 16.0^”^ W 002^o^ 30^’^ 52.1^”^

Location: Nyoli - Wa West

Vegetation: Guinea Savanna

Site: Upper Slope

Parent Material: Cape Coast Granite

Drainage: Moderately Well Drained

Classification: Ferric Luvisol

**Table 4.** Physical and chemical analysis of the soil profile pit.

Lab. No.	Site	Horizon (cm)	pH	Org. C	Total N	Available p	Exchangeable Cations cmol/kg				T.E.B	Exch. Acidity	E.C.E.C	Base Sat.	Particle - Size Analysis			
				%	%	mg/kg	Ca	Mg	K	Na	cmol/kg	cmol/kg	cmol/kg	%	Sand (%)	Silt (%)	Clay (%)	Texture
1	Kojokpere	0 - 19	6.7	1.19	0.10	3.95	5.61	1.34	0.37	0.04	7.36	0.10	7.46	98.66	67.95	30.05	2	Sandy Loam
2	"	19 - 44	6.51	0.5	0.04	0.88	3.6	0.67	0.16	0.05	4.48	0.10	4.58	97.82	73.09	24.91	2	Loamy Sand
3	"	44 - 88	6.5	0.19	0.02	0.15	2.67	0.53	0.10	0.04	3.34	0.10	3.44	97.09	75.41	22.59	2	Loamy Sand
4	"	88 - 121	6.32	0.15	0.01	0.56	2.4	0.27	0.12	0.07	2.86	0.10	2.96	96.62	74.23	23.77	2	Loamy Sand
5	"	121 - 172	6.33	0.11	0.01	0.11	4.81	1.34	0.18	0.17	6.5	0.10	6.6	98.48	68.03	27.97	4	Sandy Loam
6	Dondori	0 - 15	5.75	0.3	0.03	2.31	1.6	0.27	0.08	0.02	1.97	0.35	2.32	84.91	63.92	34.08	2	Sandy Loam
7	"	15 - 32	5.47	0.23	0.02	1.7	1.34	0.4	0.05	0.03	1.82	0.45	2.27	80.18	48.79	47.21	4	Sandy Loam
8	"	32 - 91	5.95	0.19	0.02	1.23	3.2	1.07	0.13	0.06	4.46	0.20	4.66	95.71	35.32	46.68	18	Loam
9	"	91 - 133	5.48	0.19	0.02	0.56	3.2	1.07	0.13	0.07	4.47	0.45	4.92	90.85	24.14	49.86	26	Silty Loam
10	"	133 - 180	5.54	0.11	0.01	0.15	3.47	1.60	0.19	0.07	5.33	0.42	5.75	92.7	29.44	52.56	18	Silty Loam
11	Nyoli	0 - 12	6.03	1.22	0.11	13.95	8.81	0.80	0.23	0.08	9.92	0.10	10.02	99	59.5	38.5	2	Sandy Loam
12	"	12 - 32.	6.49	0.73	0.06	6.69	5.07	2.40	0.13	0.07	7.67	0.10	7.77	98.71	57.32	36.68	6	Sandy Loam
13	"	32 - 65	6.22	0.57	0.05	2.94	5.87	1.20	0.19	0.08	7.34	0.10	7.44	98.66	49.66	40.34	10	Loam
14	"	65 - 82	5.93	0.42	0.04	0.88	6.14	2.27	0.26	0.1	8.77	0.20	8.97	97.77	42.9	45.1	12	Loam
15	"	82 - 120	6.07	0.3	0.03	0.82	7.34	3.87	0.34	0.09	11.64	0.10	11.74	99.15	38.2	51.8	10	Silty Loam

C – Carbon, N – Nitrogen, P – Phosphorus, Ca – Calcium, Mg – Magnesium, K – Potassium, Na – Sodium, TEB – Total exchangeable bases, ECEC – Effective cation exchange capacity, sat – saturation.

## 3 Experimental design, materials, and methods

Soil profile pits were dug for the soil classification. The different layers were identified and soil samples collected from each layer for laboratory analysis. The soil color was determined using the Munsell color chart. The soil samples from the different layers were analyzed in the laboratory using standard laboratory protocols. Soil pH was determined using a glass electrode pH meter in a 1:1 soil to distilled water (soil: water) ratio, available P by the Bray and Kurtz (Bray P-1) method [3] while the modified Walkley and Black procedure as described by Nelson and Sommers [4] was used to determine organic carbon. Total nitrogen was determined using the macro Kjeldahl method [5] and 1.0 *N* ammonium acetate (NH_4_OAc) extract was used for exchangeable bases. Exchangeable acidity (hydrogen and aluminium) was determined in 1.0 *N* potassium chloride (KCl) extract [6]. Exchangeable bases were extracted using 1.0 *N* ammonium acetate. Potassium and sodium in the soil extract were determined by flame photometry using standard solutions. Effective cation exchange capacity was calculated by the sum of exchangeable bases (Ca, Mg, K, and Na) and exchangeable acidity (Al and H). Percent base saturation was calculated from the sum of exchangeable bases as a percent of the ECEC of the soil.

### 3.1 Field experiment

The plot size was 24 m^2^, with a planting distance of 60 cm × 20 cm. Asontem cowpea variety obtained from the Crop Research Institute, Kumasi Ghana was used. It was a fractional factorial experiment laid out in a Randomized Complete Block Design (RCBD) with ten treatments and four replications giving a total of 40 plots. The treatments were as follows: N-P_2_O_5_-K_2_O (Kg ha^−1^) corresponding to: 0 – 0 – 0 (control), 0 – 60 – 60, 15 – 60 – 60, 30 – 60 – 60, 60 – 60 – 60, 60 – 0 – 60, 60 – 30 – 60, 60 – 60 – 0, 60 – 60 – 30, 45 – 60 – 60.

The fertilizer was spot applied at sowing using urea, triple superphosphate and muriate of potash. Glyphosate and hand weeding were deployed for weed control. Data were taken at 2-week intervals from the second week after sowing (WAS) until the sixth WAS and was stopped after flowering.

## Conflict of Interest

The authors declare no competing interest.

## Appendix Supplementary materials

Image, application 1
